# The Role of Fine Needle Aspiration Cytology in the Diagnosis of Gallbladder Cancer: A Systematic Review

**DOI:** 10.3390/diagnostics11081427

**Published:** 2021-08-06

**Authors:** Georgios D. Koimtzis, Christopher G. Chalklin, Eliot Carrington-Windo, Mark Ramsden, Leandros Stefanopoulos, Christoforos S. Kosmidis

**Affiliations:** 1 Cardiff Transplant Unit, University Hospital of Wales, Cardiff and Vale University Health Board, Cardiff CF14 4XW, UK; chris.chalklin@gmail.com (C.G.C.); ecarrington-windo@outlook.com (E.C.-W.); markramsden@hotmail.com (M.R.); 2 Lab of Computing, Medical Informatics and Biomedical Imaging Technologies, Aristotle University of Thessaloniki, 54124 Thessaloniki, Greece; lstefano@auth.gr; 3 3rd Surgical Department, University Hospital of Thessaloniki AHEPA, Aristotle University of Thessaloniki (AUTH), 54621 Thessaloniki, Greece; dr.ckosmidis@gmail.com

**Keywords:** gallbladder, cancer, diagnosis, fine needle aspiration cytology

## Abstract

Gallbladder cancer is the most common malignancy of the biliary tract. When diagnosed in an advanced stage it has a very poor prognosis. Therefore, early diagnosis and thorough assessment of a suspicious gallbladder polyp is essential to improve survival rate. The aim of this systematic review is to assess the role of fine needle aspiration cytology (FNAC) in the management of gallbladder cancer. For that purpose, a systematic review was carried out in the MEDLINE, EMBASE, Cochrane, Scopus and Google Scholar databases between 1 July 2004 and 22 April 2021. Six studies with 283 patients in total were included. Pooled sensitivity and specificity of FNAC were 0.85 and 0.94, respectively, while the area under the calculated summary receiver operating characteristic (SROC curve (AUC) was 0.98. No complications were reported. Based on the high diagnostic performance of FNAC in the assessment of gallbladder masses, we suggest that every suspicious mass should be evaluated further with FNAC to facilitate the most appropriate management.

## 1. Introduction

Biliary tract cancer (BTC) is a rare type of malignancy that makes up of less than 1% of human malignancies and 10–15% of all primary liver cancers [1]. Most commonly, it presents in the seventh decade of life with a small predominance in males [1]. Gallbladder cancer (GBC) is the most common malignancy of the biliary tract, representing 80–95% of that group, and is the 5th most common cancer of the gastrointestinal system [2]. It has a low incidence in Western Europe and the USA but remains a significant health problem in other parts of the world, such as Central and Eastern Europe and other countries like India and Chile [1]. Gallbladder cancer typically presents either via histological workup following simple cholecystectomy, or symptomatically, usually at an advanced stage [1]. It can also present as an incidental mass on imaging. Symptoms are characteristic of gallbladder and biliary tract pathology and include right upper quadrant and epigastric pain, jaundice, nausea and vomiting, anorexia and weight loss [3]. Only 3–8% of patients have a palpable mass at diagnosis [3]. Patients with advanced disease have very poor prognosis, making early diagnosis of paramount importance. Five-year survival rates of 95–99% have been reported when diagnosis is made at stage I or II. However, this drops to 2–12% in cases where the diagnosis of malignancy is made at stage III or IV [4,5]. In general, the prognosis of gallbladder cancer is inferior to all other types of cholangiocarcinoma if it is not diagnosed at an early stage [1]. These figures demonstrate the importance of exploring all possible avenues and of applying advanced diagnostic modalities with high accuracy to achieve early diagnosis of gallbladder cancer to ensure maximum survival.

The main modality currently in use for imaging of gallbladder cancer is conventional ultrasonography [6]. It can reliably detect advanced gallbladder cancer with a sensitivity of 85% and an overall accuracy of 80%. However, it is less successful in detecting abnormalities in early stage, especially if the neoplasm is sessile or associated with gallstones [7]. Other diagnostic techniques have been developed with improved diagnostic accuracy over ultrasound for gallbladder cancer. Alternative ultrasound techniques can improve diagnostic accuracy, such as high-resolution contrast-enhanced ultrasound which can identify up to 70–90% of polypoid gallbladder lesions [8]. Endoscopic ultrasound is also widely used for further assessment of suspicious gallbladder lesions and for staging of gallbladder cancer [6]. Overall accuracy in differentiating neoplastic from non-neoplastic masses has been reported as 91.9% [6]. Endoscopic ultrasound also enables the acquisition of a tissue diagnosis via endoscopic ultrasound guided fine needle aspiration (EUS-FNA). Current guidelines for gallbladder tumors offer differing recommendations on the diagnosis of gallbladder cancer. The European Society of Medical Oncology (ESMO) guidelines for biliary cancer cite magnetic resonance imaging (MRI) with magnetic resonance imaging cholangio-pancreatography (MRCP) and contrast enhanced and diffusion weighted imaging as the best diagnostic imaging tool for gallbladder cancer, while (computed tomography (CT) is not considered as helpful [1]. For tissue diagnosis Endoscopic Retrograde Cholangio-Pancreatography (ERCP) biopsy is advised, although endoscopic ultrasound fine needle aspiration (EUS-FNA) can be used if this is negative or inconclusive [1]. On the other hand, National Comprehensive Cancer Network (NCCN) guidelines suggest CT/MRI with intravenous contrast for further imaging in patients with a suspicious mass on initial examination (usually ultrasonography) or presenting symptomatically with jaundice. In unresectable disease tissue diagnosis is recommended, and in such cases via biopsy [9]. Finally, an algorithm for managing gallbladder polyps proposed by Elmasry et al. advises assessing risk factors including Indian ethnicity, coexisting gallstones, cholecystitis, age over 60, symptomatic gallbladder polyps and single gallbladder polyps. These are assessed in combination with size of the polyp, and a decision is then made if the patient undergoes endoscopic ultrasound assessment and then potential cholecystectomy, follow up with ultrasound imaging, or is discharged from the service [4].

Despite the existence of the above-mentioned guidelines and the overabundance of publications on the diagnosis and management of gallbladder polyps and GBC, there are still some controversial matters. The most important of them is the management of a T1b GBC diagnosed on pathologic review. The latest NCCN guidelines clearly state that every tumor T1b or greater should be treated, if resectable, with extended cholecystectomy, which involves hepatic resection of segments IV B and V, lymphadenectomy and potential bile duct excision in case of malignant involvement [9]. The ESMO guidelines propose an almost identical approach, but advise considering cystic duct margin, grade, involvement of resected lymph nodes and vascular and/or perineural invasion when further resection is considered for tumors above T1a [1]. Some authors have also suggested that T1b tumors should be treated with 2–3 cm wedge resection of the gallbladder bed with lymph node dissection of the hepatoduodenal ligament [8], while others suggest that simple cholecystectomy is enough for T1b tumors less than 10 mm in size [10]. In cases of unresectable masses, current guidelines recommend further investigating of the tumor for microsatellite instability and/or mismatch repair, as well as tumor mutational burden testing [10]. In such cases, treatment options usually include systemic therapy and/or palliative chemotherapy [9]. However, even in cases of resectability, the high rates of local and distant recurrence after surgical intervention justify considering further adjuvant treatment. This usually takes the form of adjuvant chemotherapy or chemoradiation therapy, but the major limitations of the existing studies do not allow definitive conclusions [1]. Moreover, based on their increased risk for malignancy, gallbladder polyps >10 mm in diameter or between 6 and 9 mm in patients with additional risk factors (age over 50, Indian ethnicity, primary sclerosing cholangitis and/or sessile polyp) should be treated with cholecystectomy according to the current guidelines [11]. However, simple cholecystectomy is the treatment of choice only for T1a tumors, while T3 and T4 tumors are usually diagnosed preoperatively. Therefore, unexpectedly encountered T1b and T2 tumors during a cholecystectomy can cause management dilemmas, lead to incomplete oncologic resections or peritoneal metastatic disease in cases of accidental perforation of the gallbladder during surgical manipulations [8]. Moreover, the size of a gallbladder polyp has been clearly associated with an increased risk of malignancy [12], while there have been reports of advanced staged of GBC diagnosed incidentally in polypoid lesions [13]. Therefore, there is a need for more accurate pre-operative diagnosis of malignancy when suspicious gallbladder polyps are present in case a simple cholecystectomy is not enough, but more advanced surgical expertise is required to reach a better oncologic outcome with fewer complications and improved overall survival.

The aim of this systematic review is to evaluate the efficacy and safety of fine needle aspiration cytology (FNAC) in early diagnosis of gallbladder malignancy and assess the potential role it could have in the management of GBC.

## 2. Materials and Methods

This study is a systematic review that was carried out without a pre-existing registered protocol. A thorough and systematic electronic search of the literature was performed to identify articles on the diagnostic performance of FNAC on gallbladder lesions. The MEDLINE and EMBASE databases were searched from 1 July 2004 until 22 April 2021. Studies before this date were not included as they were considered to have been outdated and superseded by previous research. The following keywords and search strategy were used for the MEDLINE database:

1. Biopsy, Fine-Needle/
2. Gallbladder Diseases/ or Gallbladder/ or Gallbladder Neoplasms/
3. 1 and 2
4. limit 3 to English language,

while for the EMBASE database the following:

1. fine needle aspiration biopsy/
2. gallbladder/
3. 1 and 2
4. diagnosis/
5. 3 and 4
6. gallbladder tumor/ or gallbladder carcinoma/ or gallbladder carcinoma cell line/ or gallbladder cancer cell line/ or gallbladder cancer/
7. 1 and 6 (103)
8. 4 and 7 (14)
9. gallbladder/ or gallbladder polyp/
10. 1 and 9
11. 4 and 10
12. gallbladder disease/
13. 1 and 4 and 12
14. 1 and 12
15. cytology/
16. 1 and 2 and 15
17. 1 and 6 and 15
18. 1 and 9 and 15
19. 1 and 12 and 15
20. 5 or 8 or 11 or 14 or 16 or 17 or 18 or 19 (86)
21. limit 20 to (human and English language)

Additional search was performed using similar search strategies in the Cochrane, Scopus, and Google Scholar databases. Finally, search for grey literature was conducted on the websites of international surgical associations and networks and on available data from surgical conferences.

Two independent reviewers (E.C. and M.R.) performed the literature search on the aforementioned databases and then assessed the retrieved articles for their relevance. Initially, articles were screened based on their title and duplicate records were removed. Secondarily, titles and abstracts were examined for relevance, and any non-relevant papers were removed. Finally, full text copies of the remaining articles were obtained for further eligibility screening and subsequently for data extraction and inclusion in the qualitative and quantitative analysis. Eligibility criteria included: (1) Papers including the MESH keywords “Gallbladder Diseases” OR “Gallbladder” OR “Gallbladder Neoplasms” AND “Biopsy, fine needle”; (2) publish date post July 2004; (3) English language; (4) papers involving only adults; (5) animal models were excluded; (6) fine needle aspiration was used as a diagnostic tool for gallbladder cancer; (7) histopathological analysis and/or intra-operative findings and/or post-operative follow-up was used as a reference for diagnostic accuracy of FNA; (8) enough data was present to enable the calculation of true positive, true negative, false positive and false negative figures; and (9) studies including more than 10 patients. Each article was studied independently for data extraction. In cases of disagreement between reviewers, a third independent reviewer (CC) was involved and, ultimately, either a consensus was reached, or the majority opinion was used for the analysis.

Data extracted from each article selected for analysis included the number of patients in each study, patients’ demographics (age and sex), study design and numbers of true positive (TP), true negative (TN), false positive (FP) and false negative (FN) diagnoses by fine needle aspiration cytology. Complication rates from performing FNAC were also extracted.

Data were used to construct 2 × 2 contingency tables for each study to calculate sensitivity, specificity positive predictive value, negative predictive value, and prevalence. Pooled sensitivity and specificity, with 95% confidence intervals (CIs), were obtained. The diagnostic odds ratio (DOR) was used to compare the accuracy among the included studies. In cases where sensitivity or specificity was 100%, a value of 0.5 was added to the respective table cell in order to avoid errors in the calculations. A summary receiver operating characteristic (SROC) curve was created using the Moses and Littenberg model and a weighted area under the curve (AUC) was calculated to evaluate the diagnostic performance of FNAC. The assumption of data homogeneity is proven with the use of the Cochran Q test and the calculation of I^2^ index. Potential publication bias was also assessed by designing a funnel a plot comparing the standard error to the log(DOR) for each study. Normality of the used dataset was verified by employing the Shapiro–Wilk test, the most powerful test for the normal distribution. Data in this study are presented as mean ± standard deviation. All the statistical analyses were carried out using Reviewer Manager 5.4, “R” 4.0.4 and STATA 17 software. In the current study the value of *p* < 0.05 was used as the level of statistical significance.

This systematic review was prepared following the PRISMA checklist.

## 3. Results

**Figure 1:**
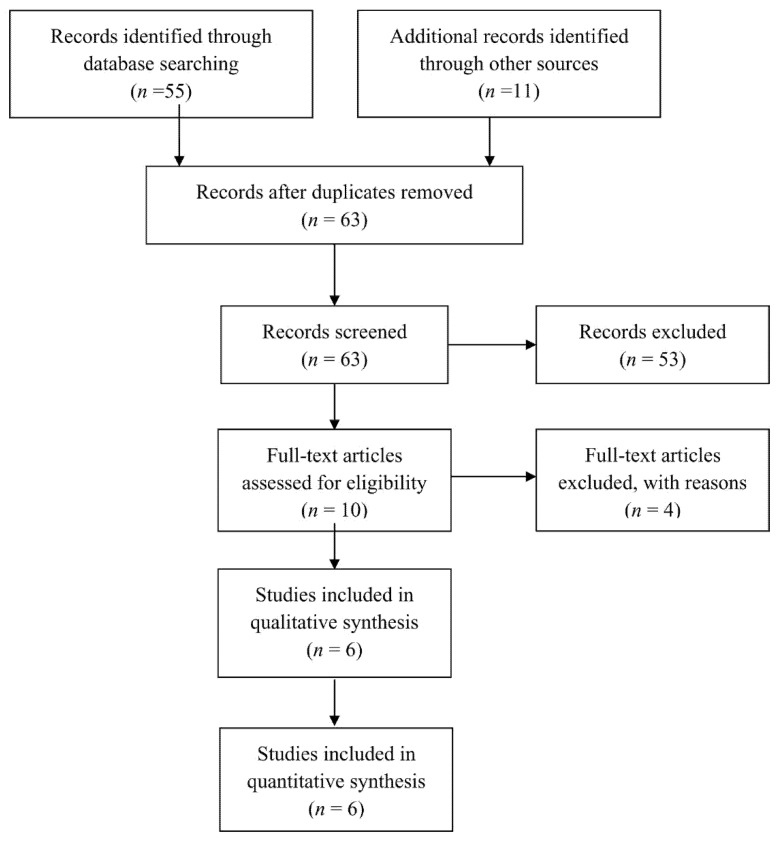
Flowchart depicting the selection process for inclusion of manuscripts in the study.

The initial search of the online databases yielded 55 articles, while the additional search of grey literature provided 11 more. Following removal of duplicate records, 63 articles were screened based on their title and abstract. This process resulted in 10 articles eligible for full-text analysis. Among them, one article was excluded due to lack of histological data on the patients, two more were excluded due to lack of clear clarification of how the final diagnosis was confirmed for every patient and finally, one article was excluded because it included only six patients. Ultimately, six articles [14,15,16,17,18,19] were selected for inclusion in the qualitive and quantitative analysis (Figure 1).

**Figure 2:**
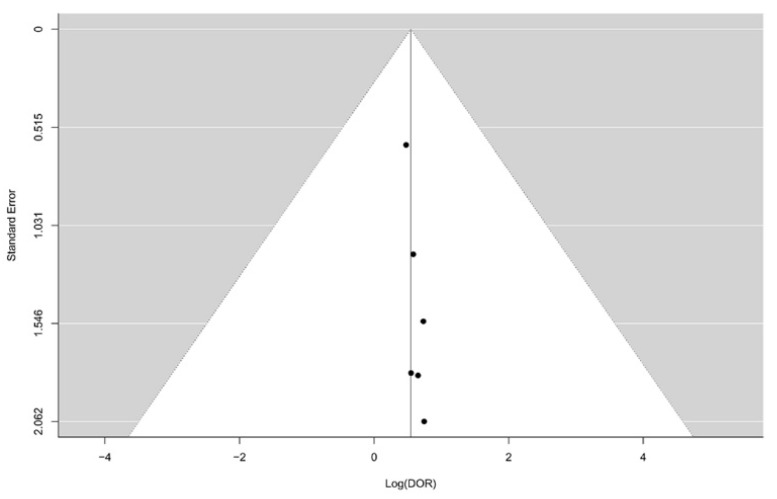
Funnel plot with pseudo 95% confidence intervals, using data from the six studies, with log-diagnostic-odds-ratios displayed in the horizontal axis, indicating that there was probably no publication bias in the included studies.

The total number of patients in the six studies included in this review was 283 (minimum: 15, maximum: 93). Four of the included studies were performed on polypoid lesions and two [15,16] on suspicious gallbladder thickening. Age and sex ratio was only mentioned in four out of the six studies. Three of the six studies were retrospective and two were prospective, while there was no mention of the study design in the final one. Sensitivity ranged from 0.64 to 1.00, while specificity ranged from 0.92 to 1.00. Positive predictive value (PPV) ranged from 0.88 to 1.00, while negative predictive value (NPV) ranged from 0.20 to 1.00. Prevalence of GBC ranged from 0.50 to 0.98, while DOR ranged from 19.94 to 256.00. Five of the six included studies reported a 0% complication rate, while one did not mention this parameter. The individual characteristics of each included study are mentioned in Table 1. The technical characteristics of how the FNAC was performed in each study are presented in Table 2. The Funnel plot designed to assess the potential publication bias in the included studies based on the log(DOR) and the standard error of each study showed symmetry indicating that there was probably no such bias (Figure 2).

**Figure 3:**
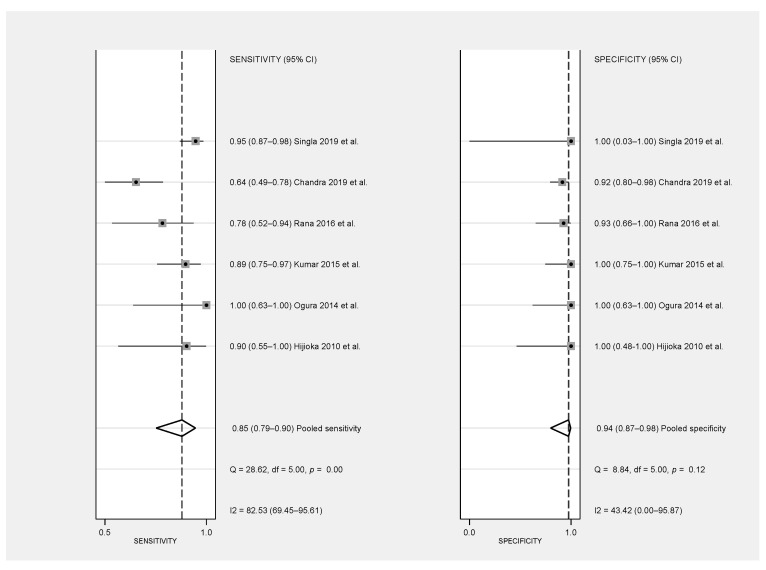
Forest plot showing study-specific [15,16,17,18,19] and pooled sensitivity and specificity with corresponding heterogeneity statistics.

**Figure 4:**
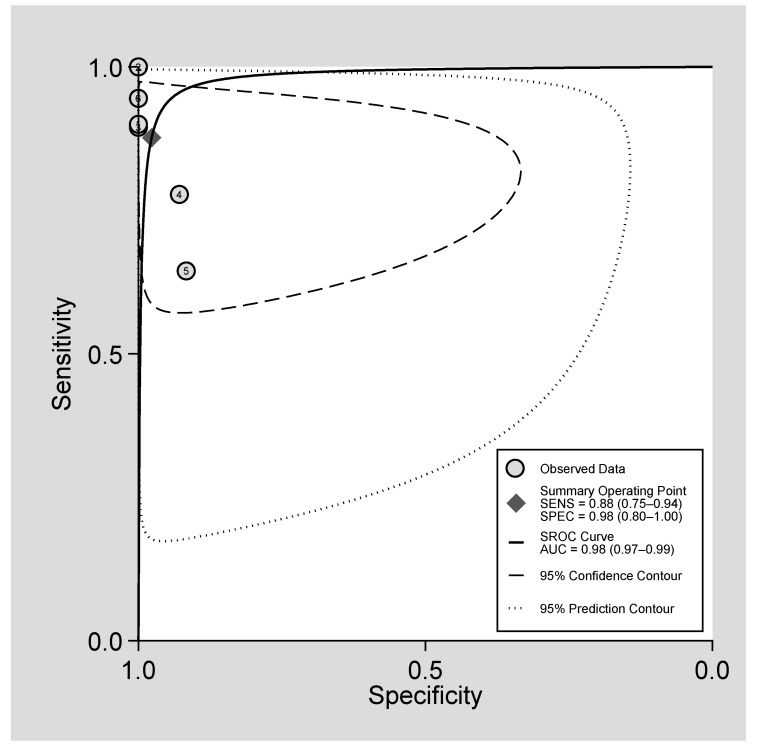
SROC curve of the performance of FNAC in the evaluation of gallbladder masses.

The pooled sensitivity of FNAC was 0.85 (0.79–0.95, 95% CI), with a large heterogeneity among studies (Q = 28.62, I^2^ = 82.53 and *p* = 0.00) and the pooled specificity was 0.94 (0.87–0.98, 95% CI) with a low heterogeneity among them (Q = 8.84, I^2^ = 43.42, *p* = 0.12) (Figure 3). The designed SROC curve yielded an AUC of 0.98 (0.97–0.99, 95% CI), indicating a very high diagnostic accuracy of the FNAC (Figure 4).

## 4. Discussion

This systematic review shows that FNAC is a diagnostic tool with very high sensitivity, specificity and diagnostic accuracy that can be very useful in diagnosing gallbladder malignancy, while it also carries a zero to minimal complication rate. Our findings confirm the results of a previous systematic review that was carried out by Wu et al. in 2011 [20]. The authors of this study reported a pooled sensitivity of 0.84 and a pooled specificity of 1.00 with low heterogeneity for endoscopic ultrasound-guided fine needle aspiration biopsy across nine studies with a total number of 284 patients. The AUC in this study was found to be 0.9254 and no complications were reported. Nonetheless, this study included both cases of gallbladder masses and distal bile duct strictures. According to our review of the literature and to the best of our knowledge, our study is the largest systematic review with the largest number of included articles and the largest total number of patients focused solely on the diagnostic performance of FNAC in the assessment of gallbladder tumors.

**Figure 5:**
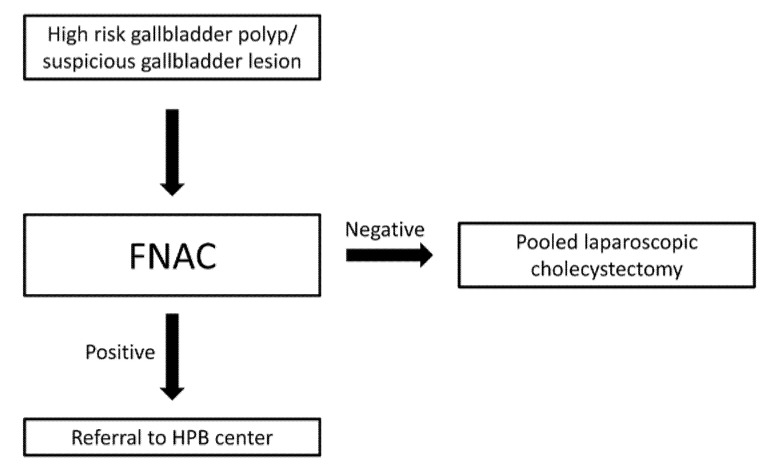
The suggested “Cardiff-Thessaloniki” modification to the management of high-risk gallbladder polyps and/or suspicious gallbladder lesions.

Despite being well-documented in the literature efficiency of FNAC in the evaluation of gallbladder malignancies, it is still not clearly included in the current guidelines of diagnosis and management of gallbladder polyps and malignancies [1,9,11]. According to these guidelines, investigating of a suspicious gallbladder mass includes multiphasic abdominal and pelvic computed tomography and magnetic resonance imaging with intravenous contrast, as well as evaluation of tumor markers, such as CEA and CA 19-9 and diagnostic laparoscopy in cases of suspicion of an irresectable tumor [9]. Biopsy is also only indicated in cases where imaging findings suggest irresectability of the tumor [9]. Moreover, currently suspicious gallbladder polyps are managed with laparoscopic cholecystectomy and post-operative histopathologic evaluation without further pre-operative investigations [11]. Laparoscopic cholecystectomy, especially when it is performed for the treatment of gallstone disease, is an operation that is usually carried out by general surgeons but is also performed by colorectal surgeons, upper gastrointestinal surgeons, hepatopancreatic biliary (HPB), oesophago-gastric, breast and vascular surgeons based on the largest United Kingdom’s audit (CholeS study) [21]. This procedure also plays an integral part in the training and development of operative skills of surgical trainees [22] as it is one of the first procedures that surgical residents gain competence in and become able to perform independently [23]. Based on the findings of our study and the importance of early recognition of gallbladder cancer and appropriate surgical approach, which requires expertise in order to avoid intraoperative perforation of the gallbladder to prevent peritoneal spreading and potentially perform a more complex operation to achieve complete oncologic resection and provide the patient with the optimal outcome and the highest survival rate possible, we propose a modification to the current guidelines of the management of gallbladder polyps and other suspicious lesions. When a patient is diagnosed with a gallbladder polyp that is symptomatic and/or deemed ‘suspicious’ according to the current criteria (size over 10 mm or size 6–9 mm and one more risk factor), or with another suspicious lesion (such as gallbladder thickening) instead of the patient being referred directly for a laparoscopic cholecystectomy, we suggest that the polyp is further investigated with FNAC. The procedure can be performed either under transabdominal or endoscopic ultrasound guidance depending on the availability and expertise of the involved center, as it carries a similar (minimal) complication rate [24,25]. If the FNAC is positive or suggestive of malignancy, then we suggest that the patient should be referred to hepatobiliary (HPB) surgeons in a center with high level of expertise in the field for further investigation and management. Otherwise, the patient can be referred for a routine laparoscopic cholecystectomy in a pooled waiting list, without further investigations required (Figure 5). However, based on the NPV of the included studies that ranged from 0.2 to 1.0 (average 0.71), there is still an almost 30% chance of patients with gallbladder carcinoma being referred to a pooled waiting list. Therefore, other groundbreaking diagnostic modalities that have not yet been included in the guidelines of management of GBC but have already shown very promising outcomes should be investigated further to facilitate a more thorough diagnostic approach of gallbladder tumors, especially in cases of negative FNAC.

High resolution ultrasonography (HRUS) offers greater diagnostic accuracy when compared to conventional ultrasound as it can visualize the layers of the gallbladder wall better [26,27]. This is attributed to the fact that conventional ultrasonography uses a low frequency transducer, which has good penetration of the abdomen but produces relatively low-resolution images. The addition of a high frequency transducer, which produces high resolution images but allows comparably less abdominal penetration, can significantly enhance the diagnostic capability of conventional ultrasound [28]. Another advanced form of ultrasonography that can be used in the assessment of gallbladder masses is the contrast-enhanced ultrasonography (CEUS) [27]. CEUS can be used for the evaluation of many intrabdominal organs. It uses a microbubble contrast which enables the exploration of tissue microvascularity, therefore overcoming the limitations of conventional ultrasonography. This contrast is safer and carries a lower risk of adverse effects when compared to the ones used for CT or MRI scans. As a result, it allows dynamic evaluation of the gallbladder, which has shown promising outcomes in differentiating between benign and malignant masses [27]. Endoscopic ultrasound is another valuable diagnostic tool even when it is not combined with FNAC but has the disadvantage of requiring sedation and can cause nausea and other mild gastrointestinal symptoms [29]. In a comparative study between HRUS and endoscopic EUS, the ability to predict gallbladder malignancy was comparable. For EUS, sensitivity was 86.2% and 86.9%, while for HRUS these were 89.6% and 86.9%, respectively [29]. In this study, both imaging modalities were compared to a conventional CT scan. Although, there was no statistically significant difference in the diagnostic accuracy for predicting the presence of malignancy and for predicting the depth of cancer invasion or differentiating between T1a and T1b tumors, HRUS and EUS were always superior when compared to CT scan [29]. A more recent study of HRUS demonstrated a sensitivity of 82.7% and a specificity of 44.4% for gallbladder cancer, with a PPV of 82.7%, a NPV of 44% and an overall accuracy of 73.6% [30]. In this study, these values were compared to the respective ones of EUS, which had a sensitivity of 86.2%, a specificity of 22.2%, a PPV of 78.1%, an NPV of 33.3% and an overall accuracy of 71%, showing that HRUS has a higher specificity PPV, NPV, as well as overall accuracy, but EUS has higher sensitivity [30]. Further studies have shown HRUS to be successful when evaluating gallbladder lesions [26,28,29]. Therefore, overall HRUS has been demonstrated to safely distinguish between benign and malignant gallbladder lesions.

Computed tomography is also one of the most commonly used diagnostic tools in the evaluation of suspected gallbladder cancer and is useful for characterizing and defining the extent of carcinoma [31]. Furthermore, fluorine-18-fluorodeoxyglucose (18F-FDG) positron emission tomography (PET), a non-invasive imaging method, is used to assess the disease extent in cancer patients. 18F-FDG is a glucose analogue and is utilized in detecting malignant lesions which usually express high glucose metabolism [32]. As 18F-FDG PET is a whole-body imaging technique, it can result in the detection of unsuspected metastatic lymph nodes or distant spread that may lead to major changes in the surgical management of patients with biliary tract cancer [33]. Hybrid PET/CT device allows enhanced detection and characterization of neoplastic lesions, by combining the functional data obtained by PET with morphological data obtained by CT [32]. In a meta-analysis of 21 studies with a total number of 495 patients performed by Annunziata et al. in 2015, results indicated that 18F-FDG PET or PET/CT have a pooled sensitivity of 87% and a pooled specificity of 78% with an AUC of 0.8787 in the evaluation of primary tumors in patients with gallbladder cancer [34]. 18F-FDG PET/CT has also an important role to play in diagnosing recurrent disease as it carries a sensitivity of 97.6% and specificity of 90% and a positive predictive value, negative predictive value, and accuracy of 95.3%, 94.7%, and 95.1%, respectively in such cases [35].

Diffusion weighted (DWI) magnetic resonance imaging (MRI) has also been suggested as an advanced diagnostic tool to differentiate between benign and malignant gallbladder lesions. In a retrospective study of 126 patients by Lee et al. in 2014, the sensitivity, specificity, PPV, and NPV of combined T2WI and DWI were 97.2%, 92.2%, 83.3%, and 98.8%, respectively. Diagnostic accuracy for gallbladder carcinoma slightly improved after adding DWI to regular MRI, from 92% to 95% with overall statistical significance, indicating that DWI can improve diagnostic accuracy for gallbladder cancer [36]. Moreover, in a meta-analysis of 8 studies including a total number of 592 patients, performed by Kuipers et al. in 2020, pooled sensitivity and specificity rates were found to be 0.87 and 0.84, respectively [37].

Advanced techniques of computed tomography have also been proven useful in the diagnostic approach of gallbladder tumors. A retrospective study of 211 patients published by Tao et al. in 2020, where a logistic regression multivariate analysis was applied showed that triphasic dynamic contrast enhanced computed tomography has an overall accuracy of 80.5%, a sensitivity of 86.7%, a specificity of 75.2%, a PPV of 75.2%, an NPV of 86.7% and an AUC of 0.875 [38]. In another study by Jindan et al. in 2018, multidetector computed tomography was found to be reliable in detecting both primary gallbladder cancer, but also assessing its local extension into the liver as well as the presence of metastatic disease to lymph nodes and distant sites [39].

The current study is limited by the fact that the included studies did not clearly mention the tumor characteristics where FNAC was performed and especially their size. Therefore, it cannot be deduced safely that FNAC can be safely carried out and be diagnostic in gallbladder tumors that are small in size. However, based on the zero complication rate that is clearly mentioned in the literature, we suggest that a prospective study on gallbladder masses up to 20 mm in size should be undertaken in order to assess the diagnostic accuracy of FNAC in such tumors and potentially strengthen the findings of this study.

## 5. Conclusions

This systematic review demonstrates that FNAC is a diagnostic tool with very high efficiency that can play a major role in the management of gallbladder masses, by facilitating accurate diagnosis of GBC. FNAC can be performed either by a regular transabdominal ultrasound or an endoscopic ultrasound with an insignificant complication rate. Based on the importance of diagnosing GBC at an early stage to ensure maximum survival, we suggested that all suspicious gallbladder polyps/masses should be further evaluated with FNAC. In case of a positive result, the patient should be referred to an advanced HPB center for further evaluation and management. Otherwise, the patient can be listed for a regular cholecystectomy in a non-urgent basis without further investigations needed. Exploration of other advanced diagnostic techniques should also be attempted in the future to propose further modifications to the current guidelines to ensure the best possible outcomes for patients with GBC.

## Figures and Tables

**Table 1:** Characteristics of each individual study included in the systematic review.

Study	Number of Patients	Age	Sex (M:F)	Study Design	Sensitivity	Specificity	PPV	NPV	Prevalence	DOR
Hijioka, et al. [14]	15	66.4 ± 12.7	7:8	Retrospective	0.9	1.00	1.00	0.83	0.67	90.00
Ogura, et al. [15]	16	70.3 ± 10.4	12:4	Not mentioned	1.00	1.00	1.00	1.00	0.50	256.00
Kumar, et al. [16]	51	Not mentioned	13:38	Prospective	0.89	1.00	1.00	0.76	0.74	221.00
Rana, et al. [17]	32	Not mentioned	Not mentioned	Retrospective	0.78	0.93	0.93	0.76	0.56	45.50
Chandra, et al. [18]	93	54.93	1:2.4	Retrospective	0.64	0.92	0.88	0.73	0.48	19.94
Singla, et al. [19]	74	58.42 ± 13.37	Not calculatable	Prospective	0.95	1.00	1.00	0.20	0.98	34.50

**Table 2:** Technical characteristics of the procedure in each study.

Study	Guidance Modality	Needle Size	Site Punctured	Puncture through Accumulated Bile	Cytology of Bile
Hijioka, et al. [14]	EUS	22 gauge	GB mass (15) and lymph node (4)	Not mentioned	Not mentioned
Ogura, et al. [15]	EUS	22 or 25 gauge	GB mass only	No	Not applicable
Kumar, et al. [16]	Not clarified	23–27 gauge	Not clarified	Not mentioned	Not mentioned
Rana, et al. [17]	Ultrasonography	22–23 gauge	GB mass only	Not mentioned	Not mentioned
Chandra, et al. [18]	Ultrasonography or CT	22 gauge	Not mentioned	Not mentioned	Not mentioned
Singla, et al. [19]	EUS	22 gauge	GB mass only	No	Not applicable

## Data Availability

The data supporting the findings of this study are available within the article.
